# Assessment of Potential Toxic Heavy Metal Levels in Serum of Saudi Patients Under Regular Hemodialysis and Its Association with Parathyroid Hormone, Uremic Pruritus, and Anemia

**DOI:** 10.3390/toxics13040241

**Published:** 2025-03-24

**Authors:** Sadyah Nedah Alrashidi, Samia Soliman Barghash, Abuzar E. A. E. Albadri, Sona S. Barghash

**Affiliations:** 1 Department of Pharmacology and Toxicology, College of Pharmacy, Qassim University, Buraydah 51452, Saudi Arabia; sadyah.rashidi@gmail.com (S.N.A.); sa.barghash@qu.edu.sa (S.S.B.); 2 Department of Forensic Medicine and Clinical Toxicology, Faculty of Medicine for Girls, Al-Azhar University, Cairo 11651, Egypt; 3 Department of Chemistry, College of Sciences, Qassim University, Buraidah 51452, Saudi Arabia; 4 Pharmaceutical Analytical Chemistry Department, Faculty of Pharmacy (Girls), Al-Azhar University, Cairo 11754, Egypt; sonamohamed1926.el@azhar.edu.eg

**Keywords:** chronic kidney disease patients, toxic elements, uremic pruritus, regular hemodialysis, parathyroid hormone, anemia, ferritin, aluminum, lead, cadmium, chromium, arsenic

## Abstract

Worldwide, environmental pollution is a major contributor to illness and mortality, encompassing toxic elements, air pollutants, agricultural pesticides, and contaminated food and water. In patients with end-stage kidney disease, several factors—including impaired renal excretion, the degree of renal impairment, medication use, dialysate contamination, the quality of dialysis water, and metabolic changes—may lead to the accumulation of toxic elements in hemodialysis patients. This study aimed to assess toxic element levels in adults undergoing hemodialysis compared to a control group and to investigate the correlation between parathyroid hormone (PTH) levels, uremic pruritus, anemia and toxic element concentrations. A cross-sectional study was conducted on 60 adult patients undergoing regular hemodialysis for at least three months. Another group of 60 apparently healthy adult voluntaries with matched age and sex with the patient group served as the control. The Inductively Coupled Plasma Mass Spectrometry (ICP-MS) method was used to measure the concentrations of serum levels of aluminum (Al), lead (Pb), cadmium (Cd), chromium (Cr), and arsenic (As) for both groups, as well as in drinking water and dialysate water. The hemodialysis group exhibited significantly higher levels of Al, Pb, Cd, Cr, and As compared to the control group. Serum Pb levels showed a significant negative correlation with PTH, while serum ferritin levels were negatively correlated with Cr. However, no significant correlation was found between toxic element levels and uremic pruritus or anemia. Toxic element concentrations in dialysis and drinking water samples were within acceptable limits and below the detection threshold set by the World Health Organization (WHO) and the Association for the Advancement of Medical Instrumentation/American National Standards Institute (AAMI/ANSI). Therefore, elevated toxic element levels in hemodialysis patients may not be primarily attributable to drinking water or dialysis.

## 1. Introduction

According to the definition of chronic kidney disease (CKD), kidney damage, or an estimated glomerular filtration rate (eGFR) of less than 60 mL/min/1.73 m^2^ for at least three months, is considered chronic. As kidney function progressively declines, kidney replacement therapy, such as dialysis or a kidney transplant, becomes necessary [1]. In patients with end-stage renal disease (ESRD), various factors influence serum trace element concentrations, including increased oral intake, impaired renal excretion, the degree of renal impairment, medication use, dialysate contamination, the quality of water used for dialysis, and metabolic changes associated with kidney failure [2].

Worldwide, environmental contamination is a major cause of illness, incapacity, and mortality. Examples include exposure to toxic elements, polluted air, agricultural pesticides, and contaminated food and water [3]. Exposure to toxic elements can lead to various severe illnesses, including cancer, neurological disorders, and respiratory diseases. Among these elements, As, Cd, Cr, Pb, and mercury (Hg) are the most common causes of poisoning in humans [4].

Hemodialysis patients who undergo dialysis three times a week are exposed to 300–600 L of water, depending on the reason for their dialysis. Additionally, based on their residual kidney function and solute clearance, hemodialysis patients are exposed to significant volumes of dialysate water. The accumulation of toxic elements is a serious concern for individuals receiving hemodialysis, as it can lead to several medical complications, including osteomalacia, low parathyroid hormone levels, anemia, increased erythropoietin requirements, dialysis-induced encephalopathy, and higher mortality rates [5]. As toxic elements enter the body, they accumulate in the brain, parathyroid glands, bones, and other organs. The accumulation of these toxic elements can lead to toxicological effects. Toxic element toxicity often accelerates cell death due to chronic disruption of cellular metabolism and may interfere with the glucose transphosphatase cycle, disrupt PTH function, impair bone metabolism, and alter essential serum elements such as calcium and phosphorus [6]. Toxic elements exert both direct and indirect effects on the parathyroid glands through various mechanisms. Their accumulation in the parathyroid glands may either enhance or diminish the parathyroid response to hypocalcemia or hypercalcemia. Additionally, toxic elements interfere with PTH synthesis and inhibit the proliferation of parathyroid cells [7].

One particularly harmful xenobiotic that causes significant health issues is Pb. Due to its properties, such as a low melting point and resistance to corrosion, lead has been widely used in various products—including paint, waterproofing tape, cosmetics, and gasoline—despite its toxicity. As a result, lead exposure has been associated with immunoregulatory, hematological, reproductive, and gastrointestinal disorders. Since the kidney is the main organ for excretion and is highly susceptible to oxidative stress and lipid peroxidation, it serves as a major site of Pb-induced toxicity. Acute Pb poisoning has been linked to proximal tubule dysfunction, as evidenced by aminoaciduria, glycosuria, and phosphaturia [8].

Cd, a hazardous toxic element originating from industrial and agricultural sources, has a detrimental impact on human health. It has been linked to cancerous tumors, bone disease, cardiovascular illness, infertility, and CKD. The accumulation of Cd in the kidneys, liver, and bones poses significant health risks. The association between low-level environmental Cd exposure and diseases such as renal disease, lung disease, hypertension, and dyslipidemia is becoming increasingly evident. Environmental sources of Cd include fuel combustion, phosphate fertilizers, smoking, contaminated food or water, and low-quality paints or cosmetics. In patients with ESRD, including those undergoing hemodialysis (HD) and peritoneal dialysis (PD), blood Cd levels appear to be correlated with an increased mortality risk [9].

One 3D transition element that occurs naturally is chromium (Cr; atomic number 24). Cr ranks as the sixth most prevalent metal in the Earth’s crust [10,11]. Cr comes in two most stable forms, trivalent (Cr III) and hexavalent (Cr VI), although it can exist in a range of oxidation states from −4 to +6. Furthermore, Cr metal, or Cr (0), is a form of Cr present in the environment. Compared to Cr (III), Cr (VI) is 100 times more toxic and significantly more soluble in water. The unstable Cr (V) and Cr (IV), along with the stable Cr (III), interact with proteins and DNA through various mechanisms, leading to DNA crosslinks and single- and double-stranded DNA breaks. The initial intracellular concentration of Cr (VI) determines the dose-dependent genetic alterations that occur [10,12]. Chromium intermediates and metabolites can induce cellular death, alter gene methylation, and disrupt histone and microRNA regulation, create microsatellite instability, and impair the function of tumor suppressor genes such as p16 and p53 [10]. Acute inhalation exposure to high concentrations of chromium (VI) has also been linked to neurological and gastrointestinal side effects, while cutaneous exposure can cause skin burns. In both humans and animals, ingestion of large doses of chromium (VI) can lead to gastrointestinal effects, including vomiting, bleeding, and stomach discomfort [13,14].

Chronic exposure to Cr (VI) in humans can cause respiratory conditions such as bronchitis, impaired pulmonary function, pneumonia, asthma, nasal irritation, and soreness, as well as septal perforations and ulcerations. Long-term inhalation or oral exposure to high levels of chromium (VI) may also impact the immune system, gastrointestinal tract, liver, and kidneys. Additionally, local exposure to chromium (VI) can result in skin irritation, ulceration, and contact dermatitis [13,15].

For optimal health, adults should consume 50 to 200 µg/day of chromium (III), which is considered an essential element for human health [10,12]. However, oral exposure to chromium (III) has been found to exhibit moderate toxicity in acute animal studies [15].

Arsenic is a metalloid and a pnictogen, sharing many characteristics with its Group 15 neighbors, antimony and phosphorus [16]. Naturally occurring metallic As is found in large quantities in the Earth’s crust and groundwater, as well as in trace levels in the air and food items, particularly seafood and shellfish. As is associated with various complications in the body’s organ systems, including the skin, nervous, respiratory, cardiovascular, hematopoietic, immune, endocrine, liver, kidney, reproductive, and developmental systems [17].

In the general population, exposure to aluminum and other toxic elements occurs through food (particularly processed foods), beverages, medications containing Al, Al cookware, and cosmetic products (e.g., antiperspirants, sunscreen, and toothpaste) [18]. The two primary routes through which toxic elements enter the human body are ingestion (through food and drink) and inhalation; however, the skin can also absorb these elements. Workers in Al-related industries face additional risks of inhalation exposure, particularly in primary metal refining, downstream sectors manufacturing Al products (such as automobiles, airplanes, and metal goods), and Al welding. Additionally, cigarette smoke can elevate the levels of toxic elements in the air [19]. Approximately 1.5% to 2% of inhaled aluminum and 0.01% to 5% of ingested Al are absorbed, with the majority being excreted in the urine. In dialysis patients, the accumulation of toxic elements may result from environmental or dietary exposure, as well as contaminated dialysate [19].

The back is the most frequently affected area from uremic pruritus (UP), though it can also involve the limbs, head, and abdomen. The distressing sensation of UP is prevalent among individuals with ESRD. It significantly impacts the quality of life of HD patients and is associated with an increased risk of mortality [20]. Despite its high prevalence, the pathophysiology of UP remains complex and poorly understood. The primary theories underlying UP include inflammation, central and peripheral nervous system dysfunction, endogenous opioid dysregulation, and impaired skin function. Additionally, factors such as iron deficiency anemia, hepatitis virus infection, xerosis, hyperparathyroidism, calcium phosphate precipitates, and other conditions contribute to the pathophysiology of UP [18].

This study aimed to assess the toxicity levels of potential toxic elements in adults undergoing regular hemodialysis compared to a control group and to evaluate the effects of Al, Pb, Cd, Cr, and As on PTH and other blood parameters. Additionally, it aimed to investigate the relationship between uremic pruritus, anemia, and toxic element levels in these individuals. To our knowledge, no similar studies have been conducted in Saudi Arabia.

## 2. Materials and Methods

### 2.1. Study Design

A cross-sectional study was conducted on 60 adult patients aged 18 and above from Buraidah Central Hospital’s hemodialysis unit. A control group of 60 healthy volunteers was also included. Medical records were analyzed for a comprehensive history, including age, sex, residency, marital status, education level, occupation, smoking habits, duration of hemodialysis, medical and medication histories, toxicological history, and clinical examinations. Levels of calcium, serum phosphate, iron profile, ferritin, total cholesterol, triglycerides, albumin, blood urea nitrogen, serum creatinine, and parathyroid hormone were obtained from the medical records.

### 2.2. Study Groups

The study involved two groups: a hemodialysis group of 60 patients undergoing regular hemodialysis with single-use hollow-fiber dialyzers fitted with modified membranes and a control group of 60 subjects from Buraidah Central Hospital.

The inclusion criteria for the hemodialysis group comprised patients who had been undergoing regular hemodialysis for more than three months, for 4 h per session, three times a week, and had controlled blood pressure and blood sugar levels. The exclusion criteria for this group included pregnant patients, cancer patients, adults with acute kidney injury, peritoneal dialysis patients, and individuals with autoimmune diseases.

The inclusion criteria for the control group comprised subjects who were apparently healthy and had controlled blood pressure and blood sugar levels. The exclusion criteria for this group included subjects with a history of cancer or autoimmune diseases.

Prior approval and informed consent were obtained from all participants.

### 2.3. Ethics Statement

The study received approval from the Saudi Ministry of Health’s College of Pharmacy and Research Ethics Committee. Participants were informed of the study’s purpose before providing their informed consent. The study was also ethically reviewed by the Qassim Region Research Ethics Committee (Approval Number: 607-45-13842; 22 April 2024).

### 2.4. Sample Collection and Preparation

Blood samples were collected from the venous port of the catheter or hemodialysis fistula before heparin was added. The samples were centrifuged to separate the serum. A microwave digestion system (Milestone, START D, Leutkirch am Allgäu, Germany) was used to digest serum samples weighing between 0.5 and 1.0 g. The digestion process involved adding 2 mL of HNO_3_ (Pure, USP, Ph. Eur., PanReac AppliChem Co., Ltd., Darmstadt, Germany) and 1 mL of H₂O₂ (USP, BP, Ph. Eur., PanReac AppliChem Co., Ltd., Darmstadt, Germany). The microwave digestion system operated in three stages: (1) A 15 min ramp to 120 °C. (2) A 15 min ramp to 200 °C. (3) A 5 min hold at 200 °C [21]. After cooling, the containers were opened, and their contents were filtered and quantitatively transferred into 15 mL volumetric flasks. The final volume was adjusted with purified water. Then, ICP-MS was used to analyze.

To calibrate the system, an ICP-MS stock-tuning solution containing Pb, Cd, Cr, As, and Al (10 µg/L) was used. Multi-element calibration standard solutions (10 µg/mL) (ICP Calibration/QUALITY Control Standard, supplied by Agilent Technologies, Palo Alto, CA, USA) were used to generate calibration curves. A dynamic reaction cell was employed to reduce polyatomic interferences. The study’s operational conditions are detailed in Table 1.

### 2.5. Drinking/Dialysis Water Sample Collection

One sample from a dialysis unit and 60 filtered water samples from the hemodialysis group had been collected. To guarantee appropriate cleanliness, the samples were kept in 200 mL polypropylene scintillation vials that had been washed three times with the appropriate water. In order to stop metal precipitation and crystallization, the samples were then acidified with 69% *w*/*v* HNO_3_.

### 2.6. Uremic Pruritus

We used the Numerical Rating Scale (NRS) to assess the severity of pruritus. The NRS is an 11-point scale that indicates the severity of an individual’s symptoms. A score of 0 represents no pruritus, 1–3 indicates mild itching, 4–6 denotes moderate itching, 7–9 signifies severe itching, and 10 represents the worst symptoms possible [22].

### 2.7. Statistical Analysis

The study used Microsoft Office Excel 2010 for graphing and SPSS version 16 for data entry and analysis. Qualitative data were presented using frequencies and relative frequencies, whereas quantitative data were represented using averages, standard deviations, and ranges. A variety of statistical tests were used, such as the Spearman rank correlation coefficient, the Kruskal–Wallis test, Fisher’s Exact Test, Pearson’s Chi-square test, Kolmogorov–Smirnov test, and independent Student’s *t*-test [23].

## 3. Results

Regarding age, Table 2 revealed that there were no statistically significant differences between the control group (mean ± SD, 56.3 ± 18.7) and the hemodialysis group (mean ± SD, 57.4 ± 17.4). In addition, there were no statistically significant differences between the two groups as regards gender, residence, education, employment, or smoking status; however, there were statistically significant disparities between the two groups as regards marital status.

Table 3 demonstrates that the mean ± SD duration of hemodialysis in the hemodialysis group was 3.7 ± 3.4 years, with a maximum of 13 years and a minimum of 3 months, which is considered equivalent to 0.25 years.

In the current study, more than half of the hemodialysis group (58.3%) experienced non-uremic pruritus. Among the cases of uremic pruritus, most reported mild symptoms (21.7%), followed by the worst imaginable symptoms (8.3%), moderate itching (6.7%), and severe itching (5%), as shown in Table 4. Table 5 shows that in the hemodialysis group, there was no statistically significant correlation between toxic elements and uremic pruritus.

Regarding the levels of toxic elements in the hemodialysis and control groups, we found that the hemodialysis group had higher levels of Al, Cd, Cr, Pb, and As compared to the control group, with a statistically significant difference (*p*-value < 0.001), as shown in Table 6.

As regards the correlation between concentrations of toxic elements in serum of haemodialysis group with age, duration of dialysis, and smoking habit, there was insignificant correlation between concentrations of heavy metals in the serum of the haemodialysis group and age, duration of dialysis, and smoking habit as in Table 7.

Regarding the correlation between parathyroid hormone, phosphorus, calcium, and toxic elements in serum among the hemodialysis group, as shown in Table 8, there was a significant negative correlation between serum lead levels and PTH in the hemodialysis group (*p*-value = 0.03 *). However, no significant correlation was observed between the levels of toxic elements and either phosphorus or calcium.

There were no statistically significant differences between the hemodialysis group’s RBC, hemoglobin, and platelets with Al, Cd, Cr, Pb, and As levels, according to Table 9.

Serum Cr and ferritin had statistically significant negative relationships, as indicated in Table 10. However, in the hemodialysis group, ferritin did not correlate to Al, Cd, Pb, or As in a statistically significant way.

Regarding the levels of toxic elements in the water samples taken from the hemodialysis unit, Table 11 demonstrates that these levels were below the normal range, according to the AAMI/ANSI maximum allowable chemical contaminant levels for dialysis water in the hemodialysis unit.

The levels of each toxic element in the drinking water samples from the hemodialysis group were within acceptable limits and, in some cases, even below the detection limit, as shown in Table 12. In accordance with WHO and AAMI/ANSI specifications, the highest Pb level was found in East Buraydah (mean ± SD: 2.7 ± 6.4), the lowest Cd level was also in East Buraydah (mean ± SD: 0.03 ± 0.02), and the highest Al level was observed in North Buraydah (mean ± SD: 33.9 ± 43.9), with no statistically significant differences.

The validation method for linearity, sensitivity, selectivity, precision, and accuracy was demonstrated effectively. The method exhibited excellent linearity for each metal, with a high correlation coefficient and a small percentage relative standard deviation, as shown in Table 13 and Table 14. For each element, the Limit of Quantification (LOQ) and Limit of Detection (LOD) were determined to be 10 times and 3.3 times, respectively, based on the (ICH) guideline formula LOD = 3.3 σ/S and LOQ = 10 σ/S, where S represents the slope of the calibration curve and σ the standard deviation of the response of intercepts. The standard deviation of the response of intercepts was calculated using the standard deviation of the y-intercepts of the regression lines [24]. The method’s selectivity was assessed by observing potential interference from drinking water and human plasma matrices, showing no interference. Intraday and inter-day precision were evaluated, and the small (% RSD) values indicate good repeatability, high accuracy, and precision of the proposed method, as shown in Table 13 and Table 14. The accuracy of the method was validated through the determination of different concentrations of pure metal samples, as presented in Table 15 and Table 16.

## 4. Discussion

As observed in our study, the biological serum samples from the hemodialysis group had higher levels of all five toxic elements than those from the control group. In the current study, the hemodialysis group consisted of 48.4% males and 51.7% females, while the control group comprised 46.7% males and 53.3% females. The gender distribution between the two groups was not statistically significantly different. Regarding age, the mean ± SD for the hemodialysis group was 57.4 ± 17.4 years, while the control group had a mean age of 56.3 ± 18.7 years; the age distributions between the two groups were not statistically significantly different. These observations are consistent with those of Hegazy et al. [25], who found no statistically significant differences in age and gender between the cases and the controls. Additionally, Siha [26] showed no statistically significant age-related differences between the exposed and control groups. In the present study, the majority of participants in both the case and control groups resided in South Buraydah (48.3% and 45%, respectively), while the lowest residency was observed in North Buraydah (15% and 12.5%, respectively), with no significant differences between the groups. Moreover, when comparing the hemodialysis and control groups, a statistically significant difference was observed in marital status. The majority of individuals in both groups were married (61.7% in the hemodialysis group and 73.3% in the control group). Additionally, no significant difference was found in occupation between the two groups; the majority of individuals in the hemodialysis group were not working (58.3%), with the lowest percentage retired (20%), while in the control group, the majority were also not working (46.7%), with the lowest percentage employed (25%).

This is in agreement with Li, J. et al. [27], who reported a statistically significant difference in marital status. However, no statistically significant variations were found between the two groups in terms of occupation and educational attainment. Al is typically eliminated by the kidneys. However, it can accumulate when renal function is significantly diminished or absent due to the reduced ability to eliminate the metal [28].

In the current study, serum Al levels in CKD patients on regular hemodialysis were significantly higher than those in the control group. The serum Al levels were 187 ± 145 ppb in the hemodialysis group and 64.2 ± 35.9 ppb in the control group, with a statistically significant difference (*p* = 0.001). Our results are consistent with those of Hegazy A. et al. and Anees et al. [25,29], who reported significantly higher serum Al levels in hemodialysis patients compared to controls.

Available data in the current study indicated that the serum concentrations of Cd, Cr, Pb, and As in the hemodialysis group (with mean ± SD: 1.2 ± 1.2, 1.06 ± 2.24, 4.3 ± 2.7, and 1 ± 0.4, respectively) were higher than those in the control group (with mean ± SD: 0.1 ± 0.12, 1.3 ± 1.4, 0.002 ± 0.021, and 0.7 ± 0.4, respectively), with strong statistically significant differences (*p*-value < 0.001). Our results are consistent with those obtained by Covic, A., and Gusbeth-Tatomir, P. [30], who reported that hemodialysis patients had lower levels of selenium, zinc, and manganese, but higher levels of Cd, Cr, copper, Pb, and vanadium compared to controls. Additionally, Shanmugam, L. et al. [31] found that the mean Cr level in the ESRD group was 5.99 ± 2.74 μg/L, whereas the control group had a mean level of 1.27 ± 0.77 μg/L, with a statistically significant difference (*p* < 0.001). Zima, T. et al. [32] also observed similar findings, with dialysis patients having a mean Cr level of 3.67 ± 0.35 μg/L, which was statistically significant. Higher Cr levels are associated with the development of cancer, contact dermatitis, and hemolysis. Furthermore, Shanmugam, L. et al. [31] reported that the serum As level in the ESRD group was 4.76 ± 2.29 μg/L, and the mean Pb level was 66.56 ± 36.62 μg/L, both of which were significantly higher (*p* = 0.001) than the control group’s mean levels of 43.5 ± 24.7 μg/L. The control group’s mean As level was 1.61 ± 0.99 μg/L, which was statistically significant (*p*-value ≤ 0.001).

In our study, smoking prevalence was observed to be 16.7% in the hemodialysis group and 25% in the control group, while non-smokers represented 83.3% of the hemodialysis group and 75% of the control group. There was no significant difference between the hemodialysis and control groups regarding smoking habits.

Our findings align with those of Bandeira et al. [33], who conducted an analysis of Pb, Co, Mg, As, Cr, and Cd levels in three groups: smokers with cancer, non-smokers with cancer, and non-cancer smokers. They observed no significant differences in the quantification of metals, except for Co levels, where a significant difference was found (*p* = 0.022), with higher mean Co levels in non-smokers with cancer.

Additionally, the current study reported no significant correlation between toxic elements levels and age, duration of dialysis, or smoking habits. This contrasts with the findings of Siha et al. [26], who reported statistically significant positive correlations between serum Al levels, duration of exposure, and smoking. These differences may be explained by evidence suggesting that prolonged work exposure increases Al levels, which then accumulate in tissues and blood, increasing the overall load on the body. Furthermore, our study disagrees with the findings of Mansouri et al. [34], who investigated the effects of secondhand and active smoking on the concentration of various essential and toxic metals in breast milk. They found significant differences in the levels of As in the breast milk of active and non-smoker groups, and that the breast milk of both passive and active smokers contained significantly higher levels of Cd, Pb, and Hg than that of non-smokers. Although these levels can vary greatly across the many cigarette brands available in this country, Viana et al. [35] also showed that carcinogenic heavy metals (Pb, Cd, As, Ni, and Cr) represent a significant source of exposure and risk for both active and passive smokers.

There is a limited number of published studies on the association between PTH and toxic elements in hemodialysis patients. The current study investigates this relationship, and a correlation analysis (Spearman’s rank correlation) was conducted to test for the presence of correlations between serum levels of Al, Cd, Cr, Pb, and As with serum PTH. The findings indicated a substantial negative correlation between Pb and PTH, with a *p*-value of 0.03 and a Spearman rank of r = −0.29. Nevertheless, there was no discernible relationship between PTH and other harmful components. These results agree with those of Siha, M. [26], who reported negative correlations between serum Al levels, serum PTH, and calcium. However, their findings are in disagreement with those of Hegazy, A. et al. [25], who demonstrated a positive correlation between serum Al levels, PTH, and age. Additionally, they contrast with the work according to Sang, Z. et al. [36], who indicated that the relationship between blood levels of Pb and Cd and serum PTH in the general population was found to be non-linearly positive and negative for Pb, while there was no significant relationship between Cd and PTH in females. However, the relationship between Cd and PTH differed by gender, with all non-linearity *p*-values being less than 0.05.

Anemia affects nearly all individuals with ESRF, with the most common cause being decreased synthesis of erythropoietin (EPO) [25]. The current study revealed significant negative correlations between serum ferritin and Cr (Spearman rank r = −0.36, *p*-value = 0.01), but no correlation between ferritin and Al, Cd, Pb, and As, or between the serum levels of toxic elements and the number of RBCs, Hgb, or platelets in the blood among the case group. These results differed somewhat from those reported by Mahieu et al. [37], who studied the hemotoxic effects of aluminum intoxication in rats over six months of exposure. They found that at the first month and at the end of the study, the red cell counts significantly increased after initially declining, compared to the control group (10 ± 0.3 vs. 8.7 ± 0.2 million/mL). Therefore, although microcytic anemia may indicate long-term Al exposure, extended exposure could lead to an increase in RBC count and a recovery of hematocrit and hemoglobin concentrations.

In our study, we found that more than half of the hemodialysis group (58.3%) had non-uremic pruritus. Most pruritus patients experienced mild pruritus (21.7%), followed by the worst possible itching (8.3%), moderate itching (6.7%), and severe itching (5%). There was no statistically significant association between UP and toxic elements. This finding contrasts with research conducted by Friga et al. [38], who found a positive correlation between pruritus and serum Al levels in 94 HD patients. However, in their study of 54 HD patients, Carmichael et al. [39] did not find such a correlation. Al-containing vaccinations have been linked to chronic pruritus and itchy nodules in the general population [39]. Bergfors et al. [40] found that 77% of children with Al allergies experienced an increased incidence of itchy nodules following the administration of diphtheria-tetanus/acellular pertussis vaccinations. The interaction between pruritus and serum Al levels in individuals undergoing maintenance HD remains uncertain.

Finally, in the current study, water contaminated with toxic elements was investigated, including both waters used in the hemodialysis unit and drinking water. The findings demonstrated that the levels of toxic elements, including Al, Pb, As, Cd, and Cr, in both drinking water and dialysis water from hemodialysis centers were within permissible limits and, in some cases, even below the detection limit. This suggests that sources other than drinking water and dialysis fluid were responsible for the elevated blood concentrations of toxic elements. By meeting the WHO and AAMI/ANSI specifications, the results indicate that water quality in the hemodialysis centers was not a significant contributing factor. In contrast, several studies conducted outside Saudi Arabia, such as those by Humudat and Al-Naseri [41], revealed that dialysis fluid had very high Al content, with some also showing elevated Pb concentrations.

Previous studies have shown that the efficacy of reverse osmosis systems varies by location. Asadi et al. [42] in Qom, Taleshi et al. [43] in Yazd, and Totao et al. [44] in Italy found that the reverse osmosis system was 100% efficient in reducing the levels of the components being studied, bringing all element concentrations within acceptable ranges. However, studies conducted in Isfahan by Shahryari et al. [45], Kermanshah by Pirsaheb et al. [46], and Palestine by Abualhasan et al. [47] reported that the reverse osmosis system was inefficient in reducing the concentration of certain elements, particularly toxic ones, to acceptable levels.

This study disagrees with the findings of Hegazy et al. [25], which revealed that the amount of Al in drinking water was 300 µg/L, above the allowable limit. This was a significant finding, as Al is used as a flocculant in Egypt to purify drinking water. Al can also be found in packaging foils, beverage cans, and through the use of metal utensils for cooking. Furthermore, our findings differ from those in several regions of Pakistan, where arsenic levels in drinking water exceed the WHO-recommended threshold of 10 ppb (µg/L). According to Ashraf et al. [48], the levels of As in the three largest reservoirs in Pakistan—Lloyd (620 µg/L), Chashma (750 µg/L), and Tarbela (620 µg/L)—are higher than the WHO safe limit.

Our study aligns with Alhagri et al. [49]. They reported that the mean levels of As, Pb, Cd, Hg, Cr, and Ni are often lower in filtered water than in tap water. Furthermore, the quantities of these compounds in the water samples remained within the permissible ranges for drinking water, as evidenced by the average concentrations of each element in both filtered and tap water being below the GSO and WHO standards.

All cases and control serum samples, and water samples in the current investigation exhibited good correlation coefficients (R^2^), ranging from 0.98 to 0.999. The recoveries of all elements in drinking water samples ranged from 85.88% to 117.31%, while in serum samples, the recoveries ranged from 83.89% to 113.51%. The agreement of these results demonstrates that both the quantitative element determination and the suggested mineralization process of the samples are accurate. The LOQs and LODs reflect the lowest concentrations of elements that can be determined accurately by the procedure.

## 5. Conclusions

The study found that hemodialysis patients had significantly higher serum concentrations of toxic elements compared to the control group, including Al, Cd, Cr, Pb, and As. Serum Pb levels were negatively correlated with PTH in the current investigation, but no significant correlation was found between the levels of toxic elements and UP. Additionally, the study found no meaningful relationship between the concentrations of toxic elements and platelet count, Hgb, or RBC count. However, negative correlations between ferritin and serum Cr were statistically significant. The study suggests that drinking water is not the primary source of elevated levels of toxic elements. As, medications such as Al-containing phosphate-binding agents, medication dosage and duration, and the efficiency of hemodialysis (HD) may play significant roles. Additionally, environmental factors such as cosmetics, as well as dietary intake and age, may have an effect on a patient’s trace metal burden. For example, polluted plants like rice contain large amounts of cadmium (Cd).

## 6. Limitation

The study has limitations, including being cross-sectional, single-center, and having a moderate patient population. It also lacked information on diets, cosmetics, and other heavy metal sources. The study was limited to a subset of toxic elements, excluded certain metals linked to death, and used only one blood test. Compared to bone lead, blood lead might not be a more reliable biomarker of chronic lead exposure.

## 7. Recommendation

This work recommends raising public health awareness about the risks of toxic element toxicity, routine measurement of metal levels for new hemodialysis patients, and the implementation of dialysis water quality monitoring programs. Additionally, it advocates for health education initiatives to increase public understanding of the harmful effects of toxic elements and the importance of antioxidants in the diet. Monitoring the concentrations of toxic elements in airborne particles, soil, aquatic environments, cosmetics, and food additives is also recommended. Including natural antioxidants, such as curcumin, in the diet may help combat oxidative stress. Further investigation is needed to identify the causes of metal intoxication.

## Figures and Tables

**Table 1:** Inductively coupled plasma mass spectrometry technique’s operational conditions.

ICP-MS Condition	Value
Model Name	Agilent, 7900
RF power	1550 W
Nebulizer Type	MicroMist
Sample Introduction	PeriPump
Nebulizer Gas Flow	0.900 L/min
Collision Cell Gas Flow	4.3 mL/min
Plasma Ignition Mode	Aqueous Solution
Ion Lenses Model	x-Lens

**Table 2:** Demographic characteristics of hemodialysis group (N = 60) and control group (N = 60).

Groups Variables	Hemodialysis Cases (N = 60)	Control (N = 60)	Total (N = 120)	*p*-Value ^
Age (Years) (mean ± SD)	57.4 ± 17.4	56.3 ± 18.7	56.8 ± 18	0.725
**Sex (%)**				
Male	29 (48.4%)	28 (46.7%)	57 (47.5%)	0.855
Female	31 (51.7%)	32 (53.3%)	63 (52.5%)	
**Residency (%)**				
South Buraydah	29 (48.3%)	27 (45.0%)	56 (46.7%)	0.578
North Buraydah	9 (15.0%)	15 (12.5%)	24 (20.0%)	
West Buraydah	10 (16.7%)	8 (13.3%)	18 (15.0%)	
East Buraydah	12 (20.0%)	10 (16.7%)	22 (18.3%)	
**Marital Status (%)**				
Single	11 (18.3%)	8 (13.3%)	19 (18.8%)	0.040 *
Married	37 (61.7%)	44 (73.3%)	81 (67.5%)	
Widow	11 (18.3%)	3 (5.0%)	14 (11.7%)	
Divorced	1 (1.7%)	5 (8.3%)	6 (5.0%)	
**Educational level (%)**				
Illiterate	32 (53.3%)	26 (43.3%)	58 (48.3%)	0.285
Primary	7 (11.7%)	6 (10.0%)	13 (10.8%)	
Secondary	6 (10.0%)	4 (6.7%)	10 (8.3%)	
Tertiary	5 (8.3%)	11 (18.3%)	16 (13.3%)	
Diploma	3 (5.0%)	1 (1.7%)	4 (3.3%)	
Bachelor	6 (10.0%)	12 (20.0%)	18 (15.0%)	
Postgraduate	1 (1.7%)	0 (0.0%)	1 (0.8%)	
**Occupation (%)**				
Not working	35 (58.3%)	28 (46.7%)	63 (52.5%)	0.410
Employee	13 (21.7%)	15 (25.0%)	28 (23.3%)	
Retired	12 (20.0%)	17 (28.3%)	29 (24.2%)	
**Smoking Status (%)**				
Non-smoker	50 (83.3%)	45 (75.0%)	95 (79.2%)	0.261
Smoker	10 (16.7%)	15 (25.0%)	25 (20.8%)	

^ Chi-square, Fisher exact and independent *t*-test were used. * Significant = *p*-value < 0.05; SD = standard deviation.

**Table 3:** Duration of hemodialysis among the hemodialysis group (N = 60).

Duration of Hemodialysis (Years)	
(mean ± SD)	3.7 ± 3.4
Range (Minimum–Maximum)	12.75 (0.25–13)

SD: standard deviation.

**Table 4:** Numerical score rate of uremic pruritus among the hemodialysis group (N = 60).

Numerical Score Rate	N.	%
0 (no pruritus)	35	58.3%
1–3 (mild itching)	13	21.7%
4–6 (moderate itching)	4	6.7%
7–9 (severe itching)	3	5.0%
10 (worst imaginable symptoms)	5	8.3%

**Table 5:** Spearman rank correlation between uremic pruritus and toxic elements in serum among the haemodialysis group (N = 60).

Toxic Elements in Serum	Uremic Pruritus	
	Spearman Rank r	*p* Value
Al (ppb)	0.03	0.84
Cd (ppb)	−0.11	0.42
Cr (ppb)	0.21	0.1
Pb (ppb)	−0.15	0.25
As (ppb)	−0.03	0.83

ppb = parts per billion.

**Table 6:** Comparison between hemodialysis group (N = 60) and control group (N = 60) regarding concentration of serum toxic elements.

Groups Variables	Hemodialysis Cases (N = 60)	Control (N = 60)	*p*-Value ^
**Al (ppb)**			
Mean ± SD	187 ± 145	64.2 ± 35.9	<0.001 *
Range (Min-Max)	866 (44.1–910)	264 (4.83–269)	
**Cd (ppb)**			
Mean ± SD	1.2 ± 1.2	0.1 ± 0.12	<0.001 *
Range (Min-Max)	8.5 (<LOD-8.5)	0.5 (<LOD-0.5)	
**Cr (ppb)**			
Mean ± SD	1.06 ± 2.24	1.3 ± 1.4	<0.001 *
Range (Min-Max)	14.2 (<LOD-14.2)	8.7 (0.1–8.8)	
**Pb (ppb)**			
Mean ± SD	4.3 ± 2.7	0.002 ± 0.021	<0.001 *
Range (Min-Max)	17.7 (0.4–18.1)	0.2 (<LOD-0.2)	
**As (ppb)**			
Mean ± SD	1 ± 0.4	0.7 ± 0.4	<0.001 *
Range (Min-Max)	2 (0.5–2.5)	1.7 (0.2–1.9)	

^ Mann–Whitney U test was used. * Significant *p*-value < 0.05; SD = standard deviation; ppb = parts per billion, LOD = limit of detection.

**Table 7:** Spearman rank correlation between concentrations of toxic elements in serum of haemodialysis group (N = 60) with age, duration of dialysis, and smoking habit.

Toxic Elements in Serum	Age (Years)		Duration of Dialysis (Years)		Smoking Habit	
	Spearman Rank r	*p* Value	Spearman Rank r	*p* Value	Spearman Rank r	*p* Value
Al (ppb)	0.14	0.29	−0.19	0.14	0.06	0.64
Cd (ppb)	0.21	0.12	0.05	0.71	−0.09	0.49
Cr (ppb)	−0.11	0.42	0.11	0.43	−0.13	0.34
Pb (ppb)	0.13	0.32	−0.01	0.93	−0.22	0.1
As (ppb)	−0.1	0.45	−0.24	0.07	0.24	0.06

ppb = parts per billion.

**Table 8:** Spearman rank correlation between parathyroid hormone, phosphorus, and calcium with serum toxic elements among haemodialysis group (N = 60).

Toxic Elements in Serum	Parathyroid Hormone (pg/mL)		Phosphorus (mmol/L)		Calcium (mmol/L)	
	Spearman Rank r	*p* Value	Spearman Rank r	*p* Value	Spearman Rank r	*p* Value
Al (ppb)	0.11	0.43	0.07	0.62	−0.18	0.18
Cd (ppb)	−0.08	0.54	−0.13	0.34	0.16	0.22
Cr (ppb)	0.1	0.46	0.11	0.41	0.09	0.5
Pb (ppb)	−0.29	0.03 *	−0.22	0.09	0.00	0.98
As (ppb)	0.04	0.8	0.19	0.15	−0.2	0.12

* Significant *p*-value < 0.05; ppb = parts per billion, pg/mL = picograms per milliliter, mmol/L = millimoles per lite.

**Table 9:** Spearman rank correlation between RBC, hemoglobin, and platelets with serum toxic elements among the haemodialysis group (N = 60).

Toxic Elements in Serum	Red Blood Cells (million/mm^3^)		Hemoglobin		Platelets (10^3^/mm^3^)	
	Spearman Rank r	*p* Value	Spearman Rank r	*p* Value	Spearman Rank r	*p* Value
Al (ppb)	0.08	0.52	0.05	0.69	0.01	0.94
Cd (ppb)	0.13	0.34	0.07	0.59	0.24	0.07
Cr (ppb)	−0.11	0.39	−0.08	0.55	−0.06	0.64
Pb (ppb)	0.07	0.61	0.09	0.49	0.03	0.83
As (ppb)	−0.13	0.34	0.05	0.7	−0.12	0.35

ppb = parts per billion, million/mm^3^ = million per cubic millimeter.

**Table 10:** Spearman rank correlation between ferritin and toxic elements in serum among the hemodialysis group (N = 60).

Toxic Elements in Serum	Ferritin (ng/mL)	
	Spearman Rank r	*p* Value
Al (ppb)	−0.18	0.18
Cd (ppb)	−0.03	0.83
Cr (ppb)	−0.36	0.01 *

* Significant *p*-value < 0.05; ppb = parts per billion, ng/mL = nanograms per milliliter.

**Table 11:** Concentration of toxic elements in water samples that are taken from the hemodialysis unit.

Toxic Elements	Normal Range According to AAMI/ANSI	Concentration (ppb)
Al	10 ppb	1.82
Cr	14 ppb	0.15
Cd	1 ppb	0.03
Pb	5 ppb	0.42
As	5 ppb	0.46

ppb = parts per billion, AAMI/ANSI = Association for the Advancement of Medical Instrumentation/American National Standards Institute.

**Table 12:** Concentration of toxic elements in the drinking water samples among the hemodialysis group.

Groups	Al (ppb)	Cd (ppb)	Cr (ppb)	Pb (ppb)	As (ppb)
	Mean ± SD	Mean ± SD	Mean ± SD	Mean ± SD	Mean ± SD
South Buraydah group (N = 36)	29.6 ± 42.7	0.04 ± 0.06	0.4 ± 0.3	0.9 ± 1.2	0.7 ± 0.2
North Buraydah group (N = 12)	33.9 ± 43.9	0.03 ± 0.03	0.4 ± 0.8	0.5 ± 0.3	0.7 ± 0.3
West Buraydah group (N = 7)	20.9 ± 25.7	0.04 ± 0.04	0.3 ± 0.5	1.2 ± 2.1	0.7 ± 0.2
East Buraydah group (N = 8)	17.2 ± 14.7	0.03 ± 0.02	0.5 ± 1	2.7 ± 6.4	0.6 ± 0.3
Kruskal–Wallis test	0.9	1	5.9	0.9	1.1
*p*-value	0.83	0.8	0.12	0.82	0.78

ppb = parts per billion; SD = standard deviation.

**Table 13:** Method validation for determination of different toxic elements in serum samples.

Name	Mass	Tune Mode	ISTD	R^2^	Precision RSD%	Units	BEC	LOD	LOQ
Al	27	He	^45^Sc [He]	0.9996	7.28	ppb	0.817	0.031	0.094
Cr	52	He	^45^Sc [He]	0.9994	8.493	ppb	0.087	0.011	0.011
As	75	He	^72^Ge [He]	0.9881	6.511	ppb	0.154	0.004	0.012
Cd	111	He	^89^Y [He]	0.9998	14.521	ppb	0.166	0.001	0.004
Pb	208	He	^209^Bi [He]	0.9996	0.489	ppb	0.552	0.019	0.056

He = Helium, R^2^ = correlation coefficients, RSD = relative standard deviation, BEC = background equivalent concentration, LOD = limit of detection in the analytical solution, LOQ = limit of quantification in the analytical solution.

**Table 14:** Method validation for determination of different toxic elements in water samples.

Name	Mass	Tune Mode	ISTD	R^2^	Precision RSD%	Units	BEC	LOD	LOQ
Al	27	He	^45^Sc [He]	0.9919	7.243	ppb	0.23	0.023	0.029
Cr	52	He	^45^Sc [He]	0.9966	4.485	ppb	0.132	0.004	0.009
As	75	He	^72^Ge [He]	0.9996	6.193	ppb	0.111	0.011	0.035
Cd	111	He	^89^Y [He]	0.9941	5.098	ppb	3.365	0.009	0.028
Pb	208	He	^209^Bi [He]	0.999	0.543	ppb	0.21	0.007	0.021

He = helium, R^2^ = correlation coefficients, RSD = relative standard deviation, BEC = background equivalent concentration, LOD = limit of detection in the analytical solution, LOQ = limit of quantification in the analytical solution.

**Table 15:** Relative standard deviation (RSD%) and average element recovery from drinking water samples.

Elements	Spike 5 ppb		Spike 10 ppb		Spike 15 ppb	
	Recovery %	RSD%	Recovery %	RSD%	Recovery %	RSD%
Al	117.31	3.55	112.44	10.62	92.23	14.62
Cr	114.34	3.2	113.11	16.4	92.44	7.97
As	89.58	11.77	109.03	3.45	97.03	14.73
Cd	95.33	0.99	100.58	0.49	100.12	0.25
Pb	117.1	2.21	106.13	5.27	85.88	1.01

ppb = parts per billion; RSD = relative standard deviation.

**Table 16:** Relative standard deviation (RSD%) and average element recovery from serum samples.

Elements	Spike 5 ppb		Spike 10 ppb		Spike 15 ppb	
	Recovery %	RSD%	Recovery %	RSD%	Recovery %	RSD%
Al	113.51	1.21	108.05	0.89	97.48	1.31
Cr	83.89	2.71	99.02	2.19	100.77	4.02
As	86.14	5.2	103.33	5.49	98.22	8.95
Cd	86.01	6.89	98.02	0.78	103.48	5.97
Pb	87.68	0.47	101.51	0.47	100.8	0.36

ppb = parts per billion; RSD = relative standard deviation.

## Data Availability

The data presented in this study are available on request from the corresponding author due to privacy.
